# Functional deciphering of the fibrillin gene family in foxtail millet reveals a non-synonymous SNP associated with carotenoid accumulation

**DOI:** 10.1186/s12870-026-09543-1

**Published:** 2026-08-11

**Authors:** Rui Huang, Yanzhen Wang, Yongxiang Xie, Haigang Wang, Xianmin Diao, Juanling Wang

**Affiliations:** 1 Center for Agricultural Genetic Resources Research, Shanxi Agricultural University, Taiyuan, 030031 China; 2 College of Agriculture, Shanxi Agricultural University, Taigu, 030801 China; 3 Key Laboratory of Sustainable Dryland Agriculture of Shanxi Province, Taiyuan, 030031 China; 4 Institute of Crop Sciences, Chinese Academy of Agricultural Sciences, Beijing, 100081 China

**Keywords:** Foxtail millet, Fibrillin, Carotenoid, Plastoglobule, Molecular marker

## Abstract

**Background:**

Carotenoids are essential phytonutrients for human health and plant adaptation. In plastids, carotenoid storage and turnover occur in plastoglobules, whose core structural components are Fibrillin (FBN) proteins. The FBN family remains uncharacterized in foxtail millet (*Setaria italica*), a nutritious C4 cereal whose grain carotenoid content strongly correlates with color. The genetic basis of carotenoid variation in foxtail millet is largely unknown.

**Results:**

We identified 12 *SiFBN* genes in foxtail millet, which were phylogenetically classified into 11 subfamilies. Expression analysis revealed three major patterns: constitutive (*SiFBN1b*), development-dependent (*SiFBN6*, *SiFBN10*), and differentially expressed between varieties (*SiFBN3*). SiFBN3 was significantly upregulated in the high-carotenoid yellow-grain variety JG21 compared to JG25, though haplotype analysis in a 152-accession panel revealed no sequence polymorphism in this gene. Furthermore, a candidate-gene association analysis in a 152-accession panel identified a natural polymorphism in SiFBN10 (A/T at position 40085873) associated with carotenoid content. The T allele (Hap2, frequency 30.3%) was strongly associated with high carotenoid accumulation across two independent years, whereas the A allele (Hap1, 69.7%) linked to low content. Both SiFBN3 and SiFBN10 were experimentally confirmed to localize to chloroplasts.

**Conclusions:**

This study suggests that carotenoid accumulation in foxtail millet grains involves multiple regulatory layers. *SiFBN3* shows differential expression between the yellow-grain variety JG21 and the white-grain variety JG25, though the genetic basis of this difference remains to be elucidated given the absence of sequence polymorphism in the 152-accession panel. In addition, a natural polymorphism in *SiFBN10* is associated with carotenoid content variation in the natural population and may contribute to phenotypic differences among accessions.

**Supplementary Information:**

The online version contains supplementary material available at https://doi.org/10.1186/s12870-026-09543-1.

## Background

Carotenoids are essential lipophilic isoprenoids in plants, functioning in photosynthesis, photoprotection, and thylakoid membrane stabilization [1, 2]. Beyond their roles in plant physiology, carotenoids provide characteristic pigmentation to flowers and fruits [3] and serve as precursors for phytohormones including abscisic acid and strigolactones [4–6]. As vital provitamin A sources for human nutrition and health [7], enhancing carotenoid content is a major breeding target in cereal crops. Plastoglobules (PGs) are lipoprotein subcompartments within plastids that function as both storage depots for hydrophobic compounds like carotenoids and active hubs for lipid and carotenoid metabolism [8–10].

The Fibrillin (FBN) family constitutes the major structural protein component of PGs [11]. By mediating PG assembly, stabilization, and metabolite sequestration, FBN proteins are pivotal regulators of carotenoid storage capacity and metabolic homeostasis [12, 13], thereby serving as key determinants of carotenoid accumulation in plant tissues. FBN proteins, first identified in pepper chromoplasts [14] are widely distributed across photosynthetic organisms [15]. Genome-wide analyses have characterized of FBN families in Arabidopsis (*AtFBN*) [16], rice (*OsFBN*) [17], tomato (*SlFBN*) [18], wheat (*TaFBN*) [19], and cucumber (*CasFBN*) [20], revealing remarkable functional diversity. For example, in carotenoid metabolism, *AtFBN6* directly activates phytoene synthase to promote biosynthesis [21], whereas *BjFBN1b* facilitates lutein ester storage [22]. Other members are involved in PG stabilization and plastid development [23–26]. Despite these advances, the genetic basis linking natural variation in FBN genes to carotenoid accumulation remains largely unknown.

Foxtail millet (*Setaria italica*), an ancient C_4_ cereal domesticated in China, is valued for its exceptional drought tolerance and nutritional quality. Its grain color serves as a visual marker strongly correlated with carotenoid content [27, 28]; yellow-grain varieties accumulate substantially higher levels than white-grain varieties, providing a powerful system for dissecting carotenoid regulation [29]. Nevertheless, the *FBN* gene family remains uncharacterized in foxtail millet, and no study to date has connected natural variation in *FBN* genes with carotenoid accumulation in any cereal crop.

Our study suggests the *FBN* gene family as a candidate regulator of carotenoid metabolism in foxtail millet. The contrasting regulatory mechanisms of *SiFBN3* and *SiFBN10* (differential expression regulation vs. coding-region polymorphism) highlight the functional diversification within this gene family. Notably, the identification of a non-synonymous SNP in *SiFBN10* with consistent effects on carotenoid content across environments provides a promising molecular marker for breeding programs. These findings not only advance our understanding of carotenoid biosynthesis regulation in cereals but also offer an immediately deployable genetic tool for nutritional enhancement of foxtail millet.

## Materials and methods

### Plant materials and growth conditions

Two foxtail millet varieties with contrasting grain carotenoid content, Jingu 21 (JG21, yellow kernel, high carotenoid) and Jiugu 25 (JG25, white kernel, low carotenoid), were used for transcriptome sequencing and targeted carotenoid profiling. JG21 was developed by the Institute of Crop Sciences, Shanxi Agricultural University, and JG25 was bred by the Jilin Academy of Agricultural Sciences. Seeds were obtained from the National Medium-term Genebank for Featured Minor Cereal Crops (Taiyuan). Plants were grown under standard field conditions at the Dongyang Experimental Base of Shanxi Agricultural University (Jinzhong, Shanxi, China) from May to October 2025. The experiment followed a randomized complete block design with three biological replicates. Each plot consisted of six rows (2 m in length, with 30 cm between rows and 10 cm between plants). To capture dynamic changes during grain development, grains were sampled at 7, 14, and 21 days after anthesis (DAA). After husk removal, samples were immediately frozen in liquid nitrogen and stored at − 80 °C for subsequent RNA extraction and carotenoid analysis.

For haplotype analysis, a panel of traditional Chinese landraces was used. These materials are maintained by the National Medium-term Genebank for Featured Minor Cereal Crops (Taiyuan) and are available from the corresponding author upon reasonable request due to germplasm resource conservation policies.

### Identification and physicochemical property analysis of *SiFBN* members

The genome assembly, protein sequences and GFF3 annotation files of foxtail millet (*Setaria italica* v2.2) and *Arabidopsis thaliana* (TAIR 10) were downloaded from Phytozome (https://phytozome-next.jgi.doe.gov/) and Ensembl Plants (https://www.ensembl.org/), respectively. Protein sequences of *AtFBN* members were retrieved from the TAIR database and used as queries for BLASTP homology searches (E-value < 1^e−5^) against the foxtail millet proteome using TBtools to preliminarily identify candidate *SiFBN* genes. The hidden Markov model (HMM) profile of the FBN family conserved domain was obtained from the Pfam database and searched against the whole foxtail millet protein sequences using HMMER. All candidate sequences were validated for the integrity of FBN conserved domains through NCBI Conserved Domain Database (CDD, https://www.ncbi.nlm.nih.gov/cdd/) and SMART (http://smart.embl-heidelberg.de/), with incomplete domain sequences being excluded. Finally, *SiFBN* members were confirmed and functionally annotated through the foxtail millet genomics database. Key physicochemical properties of the identified SiFBN proteins, including amino acid number, molecular weight, theoretical isoelectric point (pI), instability index, aliphatic index and grand average of hydropathicity (GRAVY), were predicted using the ProtParam tool (https://web.expasy.org/protparam/). Subcellular localization was predicted using online tools WoLF PSORT and DeepLoc 2.0.

### Phylogenetic analysis of SiFBN proteins

To elucidate the evolutionary relationships of SiFBN proteins, FBN protein sequences of *Arabidopsis*, *Oryza* (rice), *Sorghum bicolor* (sorghum) were downloaded from the Ensembl Plants website, while FBN protein sequences of *Panicum miliaceum* (broomcorn millet) were obtained from NCBI. Multiple sequence alignment was performed using ClustalW implemented in MEGA 12.0. The phylogenetic tree was constructed by the maximum likelihood method based on the best-fit model (JTT) selected by Model Test, with 1000 bootstrap replicates to assess branch reliability. The generated phylogenetic tree was visualized and annotated using the iTOL online platform (https://itol.embl.de/).

### Analysis of gene structure, protein characteristics and cis-acting elements

The gene structures (exons, introns and UTR regions) of SiFBN members were analyzed using the Gene Structure View (Advanced) module in TBtools v2.390. Conserved motifs were identified using the online tool MEME Suite v5.0.5 (https://meme-suite.org/), with the following parameters: maximum number of motifs set to 15, motif width ranging from 6 to 50 amino acids, EM algorithm with default parameters, and 100 iterations. Promoter sequences (2 kb upstream of the start codon ATG) were extracted from the foxtail millet genome using TBtools and submitted to the Plant CARE database (https://bioinformatics.psb.ugent.be/webtools/plantcare/) for cis-acting element prediction. We focused on the analysis of elements related to light response, phytohormone response and stress tolerance. The identified elements were categorized based on their biological functions, such as plant growth and development, hormone signaling, and biotic/abiotic stress responses. The distribution patterns of these elements across the promoter regions of different *SiFBN* genes were visualized using TBtools.

### Chromosomal localization and synteny analysis of *SiFBN* family

Chromosomal location information of *SiFBNs* was extracted from the GFF3 annotation file and physical mapping was performed using TBtools v2.390. Whole‑genome synteny analyses between foxtail millet and four representative species (*Arabidopsis thaliana*, *Oryza sativa*, *Sorghum bicolor*, and *Panicum miliaceum*) were performed using MCScanX. The results were visualized with the Dual Synteny Plot tool in TBtools.

### Expression profiling of *SiFBNs* based on public transcriptome data

To investigate the expression patterns of *SiFBN* genes, publicly available RNA-seq data were retrieved from the foxtail millet expression database, covering different tissues (SetariDB-Transcriptome) and various stress treatments (https://phytozome-next.jgi.doe.gov/). Transcript abundance was quantified as TPM values. The expression matrix of *SiFBN* genes was normalized by Z-score and hierarchical clustering heatmaps were generated using TBtools to visualize gene expression patterns across different samples.

### Expression analysis of *SiFBNs* during grain development

#### RNA sequencing and expression analysis

Total RNAs were extracted using ethanol precipitation and CTAB-PBIOZOL methods. The quality and quantity of total RNA were assessed using a Qubit 4.0 fluorescence quantifier (Thermo Fisher Scientific, Waltham, USA) and a Qsep400 high-throughput biofragment analyzer (Advanced Instruments, Inc., Norwood, USA), respectively. The preparation of RNA-seq libraries and subsequent sequencing were carried out by Metware Biotechnology Co., Ltd. (Wuhan, China). FPKM values of *SiFBN* family members at different developmental stages were extracted from the sequencing results. After normalization, expression heatmaps were generated using TBtools.

#### RT-qPCR validation

RT-qPCR was performed to validate the RNA-seq results. Gene-specific primers (synthesized by Shanghai Sangon Biotech Co., Ltd., Supplementary Table S1) and SYBR Green master mix were used on a real-time quantitative PCR system. The *SiActin* gene served as the internal reference. Relative expression levels were calculated using the 2^−ΔΔCt^ method [30]. Three biological replicates were included for each treatment. Statistical significance was assessed by one-way ANOVA (*P* < 0.05).

### Carotenoid extraction and quantification

Carotenoid content was measured in accordance with the method of Li [31] for quantitative analysis. After grinding and sieving, the samples were saponified at 70 °C for 45 min using a mixture of 95% ethanol, sodium chloride (17.52 g/L), pyrogallol ethanol solution (63.055 g/L), ascorbic acid (176 mg/mL), and potassium hydroxide. This saponification step hydrolyzes esterified carotenoids; therefore, the reported values represent total carotenoids (free + liberated esterified forms). The saponified mixture was then subjected to six sequential extractions with n-hexane/ethyl acetate (9:1, v/v). Combined supernatants were evaporated under nitrogen gas and reconstituted in methyl tertbutyl ether (MTBE) containing 0.1% (w/v) butylated hydroxytoluene (BHT) for quantification.

Carotenoid analysis was performed using an Agilent 1200 HPLC system (Agilent Technologies, USA), equipped with a photodiode array detector and a YMC™ Carotenoid 30 column (5 µm particle size, 4.5 × 250 mm, Waters Corporation, Wilmington, NC, USA). The detection wavelength was set at 450 nm. Mobile phase A consisted of 90% A’ (97% methanol–water solution containing 0.05 M ammonium acetate and 0.1% BHT, w/v) and 10% B’ (MTBE containing 0.1% BHT, w/v), while mobile phase B consisted of 90% B’ and 10% A’. Gradient elution was performed at a flow rate of 1 mL/min with the following program: 0–10 min (90–60% A); 10–20 min (60–50% A); 20–25 min (50–10% A); 25–29 min (10% A); 29–29.5 min (10–90% A); 29.5–40 min (90% A). The composition of carote-noids was identified and quantified by comparing the retention times and peak areas of the samples with those of the standards using the external standard method (Sigma, Germany). Results were expressed as µg g^− 1^ dry weight (DW). All experiments were performed in triplicate.

### Subcellular localization

The full-length coding sequences of SiFBN3 and SiFBN10 (without stop codons) were amplified and cloned into the pCAMBIA1302-eGFP vector, in-frame with enhanced green fluorescent protein (eGFP) at the C-terminus, generating CaMV35S::SiFBN3-eGFP and CaMV35S::SiFBN10-eGFP constructs. The recombinant plasmids were transformed into *Agrobacterium tumefaciens* strain GV3101 and infiltrated into *Nicotiana benthamiana* leaves. After 48 h incubation, GFP fluorescence and chlorophyll autofluorescence were observed using a confocal laser scanning microscope (LSM 880, Carl Zeiss). Chloroplast targeting was confirmed by colocalization of GFP signal with chlorophyll autofluorescence. The experiment was repeated three times independently.

### Haplotype and association analysis of SiFBN10

Haplotype analysis of the SiFBN10 locus was performed on a natural population of 152 foxtail millet accessions using the R package “geneHapR” [32]. Based on the re-sequencing data, the SNP variant within the SiFBN10 coding region (chr2: 40085873, A/T) was extracted using VCFtools (v0.1.16). Two haplotypes were defined: Hap1 (A allele, *n* = 106) and Hap2 (T allele, *n* = 46). The phenotypic data (carotenoid contents: lutein, zeaxanthin, β-carotene, and total carotenoids) were matched to each accession by sample ID.

Haplotype-phenotype associations were evaluated using independent samples t-tests for normally distributed data and Mann-Whitney U tests for non-normally distributed data. Normality was assessed using the Shapiro-Wilk test. Data from 2020 to 2022 were analyzed separately. For the 2020 dataset, all four traits were analyzed using t-tests. For the 2022 dataset, lutein violated the normality assumption and was analyzed using the Mann-Whitney U test; the remaining three traits were analyzed using t-tests. All *P*-values are reported as raw (uncorrected) values; no multiple testing correction (Bonferroni or BH-FDR) was applied. Statistical analyses and visualization were performed using GraphPad Prism (v10.0).

### Statistical analysis

All statistical analyses were performed using IBM SPSS Statistics (v26.0) and R (v4.2.1). For two-group comparisons (e.g., JG21 vs. JG25, Hap1 vs. Hap2), two-tailed Student’s t-test or Mann-Whitney U test (when normality was violated) was used. For multiple comparisons (e.g., among developmental stages), one-way ANOVA followed by Tukey’s HSD test was performed. Statistical significance was defined as ^*^P < 0.05, ^**^P < 0.01, ^***^P < 0.001, and ^****^P < 0.0001. All data are presented as means ± standard deviation (SD) of three biological replicates. Figures were generated using TBtools-II (v2.390), iTOL (v6), MEME Suite (v5.5.4), GraphPad Prism (v9.0), and Adobe Illustrator (v2023).

## Results

### Identification and physicochemical characterization of *SiFBN* family

A systematic genome-wide search identified 12 *Fibrillin* (*FBN*) genes in foxtail millet (*Setaria italica* (L.) Beauv.), designated *SiFBN1a*, *SiFBN1b*, and *SiFBN2*-*SiFBN11* based on homology with *Arabidopsis AtFBNs* (Table 1). Physicochemical property predictions revealed significant variations in sequence length and charge characteristics among family members. Protein lengths ranged from 161 aa (SiFBN1a) to 643 aa (SiFBN11), with molecular weights of 17.65–71.63 kDa. Theoretical isoelectric point (pI) showed a wide distribution (4.41–9.76), with seven proteins predicted as basic (pI > 7) and five as acidic (pI < 7). Stability analysis indicated that except for SiFBN6 (instability index = 38.92), the instability indices of the other 11 members were all greater than 40, suggesting that they might be unstable proteins when purified. All members exhibited high aliphatic indices (> 75), indicating thermal stability. Grand average of hydropathicity (GRAVY) analysis identified most proteins as hydrophilic (negative values); only SiFBN1a (0.102) and SiFBN6 (0.074) were predicted hydrophobic. In silico subcellular localization prediction (WoLF PSORT and DeepLoc-2.0) consistently assigned chloroplast targeting to the 12 SiFBNs, consistent with the plastidial function of FBN proteins. Discordant predictions for SiFBN1a (chloroplast/nucleus vs. chloroplast/cytosol) may reflect ambiguous targeting signals.

**Table 1:** Basic information of *SiFBN* gene family

Name	Sequence ID	Amino Acid Number	Molecular Weight (kDa)	Theoreti-cal pI	Instabili-ty Index	Alipha-tic Index	GRAVY	Subcellular Localization	
								WoLF Resort	DeepLoc 2.0 Resort
*SiFBN1a*	*Seita2G148200*	161	17650.26	4.41	47.16	104.72	0.102	nucleus	Cytoplasm
*SiFBN1b*	*Seita2G164500*	318	33815.33	5.01	49.36	92.74	-0.095	Chloroplast	Chloroplast
*SiFBN2*	*Seita9G309700*	385	41028.83	4.53	50.08	78.36	-0.361	Chloroplast	Chloroplast
*SiFBN3*	*Seita9G124200*	258	28396.78	9.76	42.65	85.93	-0.314	Chloroplast	Chloroplast
*SiFBN4*	*Seita8G173800*	281	29702.65	8.96	45.77	84.77	-0.230	Chloroplast	Chloroplast
*SiFBN5*	*Seita7G096400*	266	28854.01	8.94	43.7	91.43	-0.211	Chloroplast	Chloroplast
*SiFBN6*	*Seita7G229800*	217	23532.38	9.15	38.92	103.27	0.074	Chloroplast	Chloroplast
*SiFBN7*	*Seita3G029400*	298	32526.25	5.74	46.23	90.77	-0.162	Chloroplast	Chloroplast
*SiFBN8*	*Seita9G195900*	317	34774.10	9.36	76.92	98.26	-0.119	Chloroplast	Chloroplast
*SiFBN9*	*Seita7G190200*	221	24226.66	6.74	44.44	90.09	-0.092	Chloroplast	Chloroplast
*SiFBN10*	*Seita2G310100*	385	42305.19	9.24	42.07	88.44	-0.169	Chloroplast	Chloroplast
*SiFBN11*	*Seita5G248500*	643	71630.45	9.36	56.62	92.36	-0.172	Chloroplast	Chloroplast

The GRAVY (grand average of hydropathicity) score reflects a protein’s overall hydrophilic or hydrophobic character: values < 0 denote a predominantly hydrophilic protein, whereas values > 0 denote a predominantly hydrophobic protein

### Phylogenetic analysis of *SiFBN* family

**Fig. 1:**
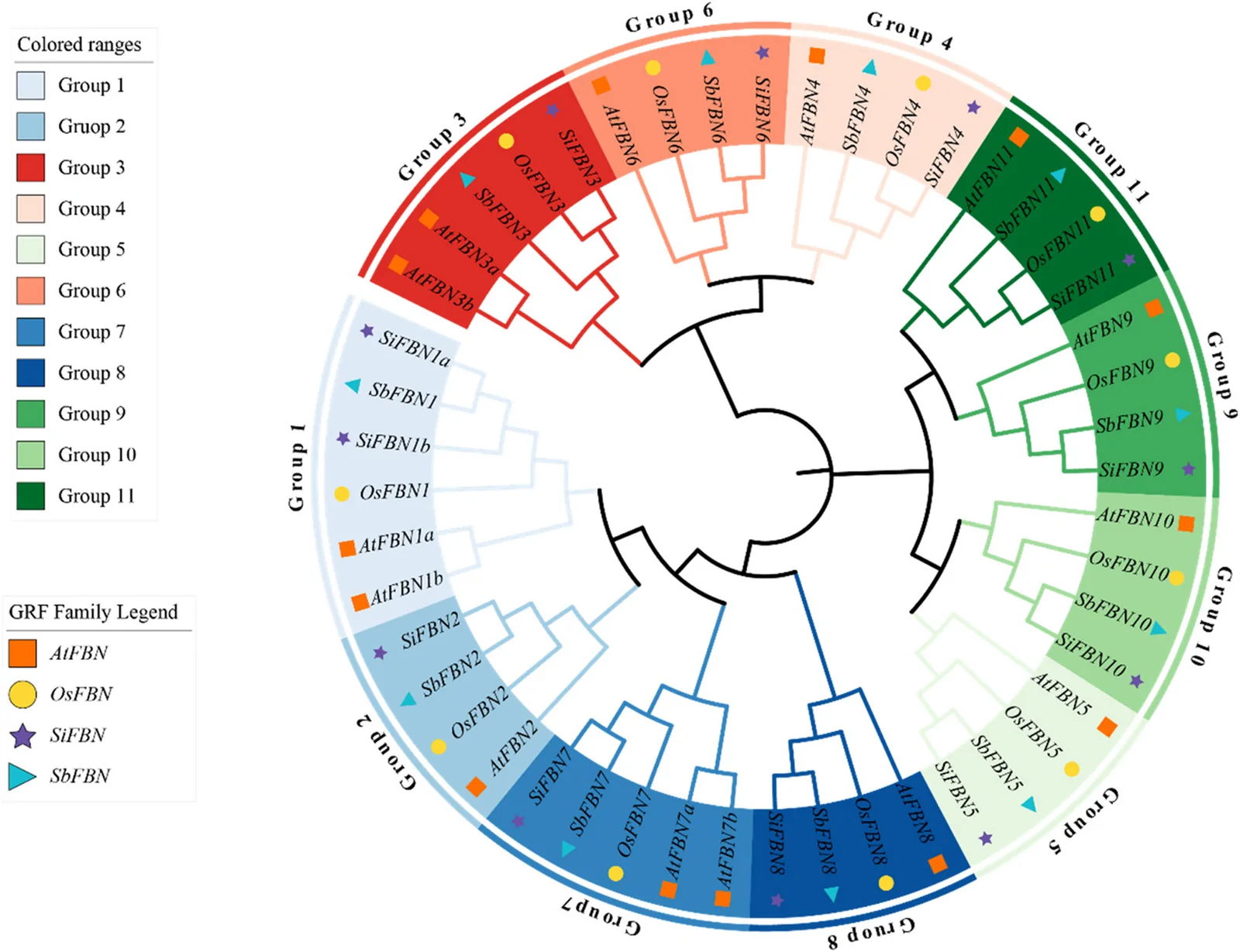
Phylogenetic tree of FBN proteins from foxtail millet (*Setaria italica*), rice (*Oryza sativa*), sorghum (*Sorghum bicolor*), and *Arabidopsis thaliana*. The tree was constructed using the maximum likelihood method with 1000 bootstrap replications. Different colored branches indicate distinct clades

To resolve evolutionary relationships, a maximum-likelihood phylogenetic tree was constructed using full-length FBN protein sequences of *Setaria italica* (12*SiFBN*), *Arabidopsis thaliana* (14*AtFBN*), *Oryza sativa* (11*OsFBN*), and *Sorghum bicolor* (11*SbFBN*). The resulting tree grouped the 48 FBN proteins into 11 clades (Group1-Group11, Fig. 1). All the 12 *SiFBN* were represented, with each occupying a unique clade.

Two paralogs, *SiFBN1a* and *SiFBN1b*, clustered tightly within Group 1, indicative of a recent gene duplication event specific to foxtail millet. The remaining 10 *SiFBNs* were distributed singly across Groups 2–11, indicating that early gene duplication events and subsequent significant functional differentiation may have occurred. The topological structure of the phylogenetic tree revealed a clear monocot-specific clustering pattern. In eight clades (Group 1, 2, 5, 6, 7, 8, 9, 10), *SiFBN* members showed a closer evolutionary relationship with *SbFBN* genes, suggesting that foxtail millet and sorghum may have conserved functions or shared evolutionary paths in these lineages. Conversely, in the remaining three clades (Group 3, 4, and 11), the *SiFBNs* clustered more closely with *OsFBN* genes, indicating a stronger phylogenetic affinity between foxtail millet and rice in these specific lineages.

### Analysis of conserved motifs, domains, and gene structures

**Fig. 2:**
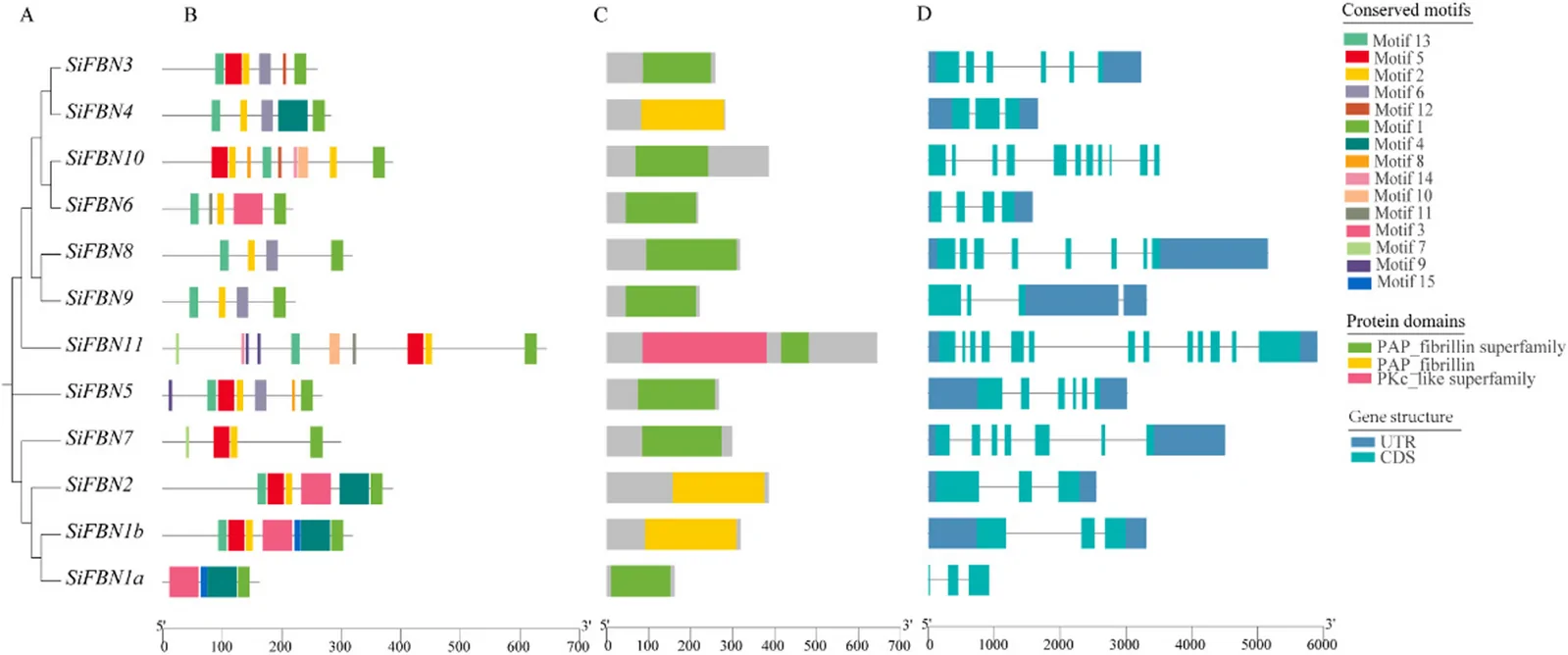
Phylogenetic relationship, conserved motifs, domains, and gene structure of *SiFBN* members. **A** Phylogenetic tree of *SiFBN* proteins. **B** Conserved motifs identified by MEME. Different colored boxes represent distinct motifs. **C** Domain architecture identified by NCBI-CDD. Different colored boxes represent conserved domains. **D** Exon-intron structures. Yellow boxes indicate coding sequences (CDS), black lines indicate introns, and blue boxes indicate untranslated regions (UTRs). Green boxes indicate upstream/downstream sequences

Conserved protein motif distribution across the 12 *SiFBNs* was analyzed using MEME, which identified 15 distinct motifs from the SiFBN coding sequences (Motif 1-Motif 15; Fig. 2B). Individual proteins contained 4–8 motifs differently among the analyzed 12 *SiFBNs*. Notably, Motif 1 was universally present and positionally conserved, suggesting it constitutes the core structural element. Motif 2 was present in 11 of the 12 proteins, being absent only in *SiFBN1a*. Several motifs exhibited clade-specific distributions: Motif 7 was exclusive to *SiFBN7* and *SiFBN11*, while Motif 15 was specific to the paralogous pair *SiFBN1a* and *SiFBN1b*. Domain analysis revealed that the defining PAP_fibrillin domain was present in all SiFBNs (Fig. 2C). Most members (11 out of 12) contained only the PAP_fibrillin family domain. Notably, *SiFBN11* carried an additional protein kinase C-like (PKc_like) superfamily domain, suggesting potential involvement in phosphorylation-mediated regulation. *SiFBN1b*, *SiFBN2*, and *SiFBN4* harbored the more specific PAP_fibrillin domain variant. Gene structure analysis revealed significant diversity in exon-intron organization (Fig. 2D). Exon numbers varied from 3 (*SiFBN1a*) to 13 (*SiFBN11*). Four genes (*SiFBN1b*, *SiFBN2*, *SiFBN4*, and *SiFBN9*) each contained five exons. Untranslated region (UTR) analysis showed that *SiFBN1a* and *SiFBN10* lacked both 5′ and 3′ UTRs, *SiFBN9* lacked the 5′ UTR, and the remaining nine genes possessed complete UTRs.

### Analysis of cis-acting elements in *SiFBN* promoters

**Fig. 3:**
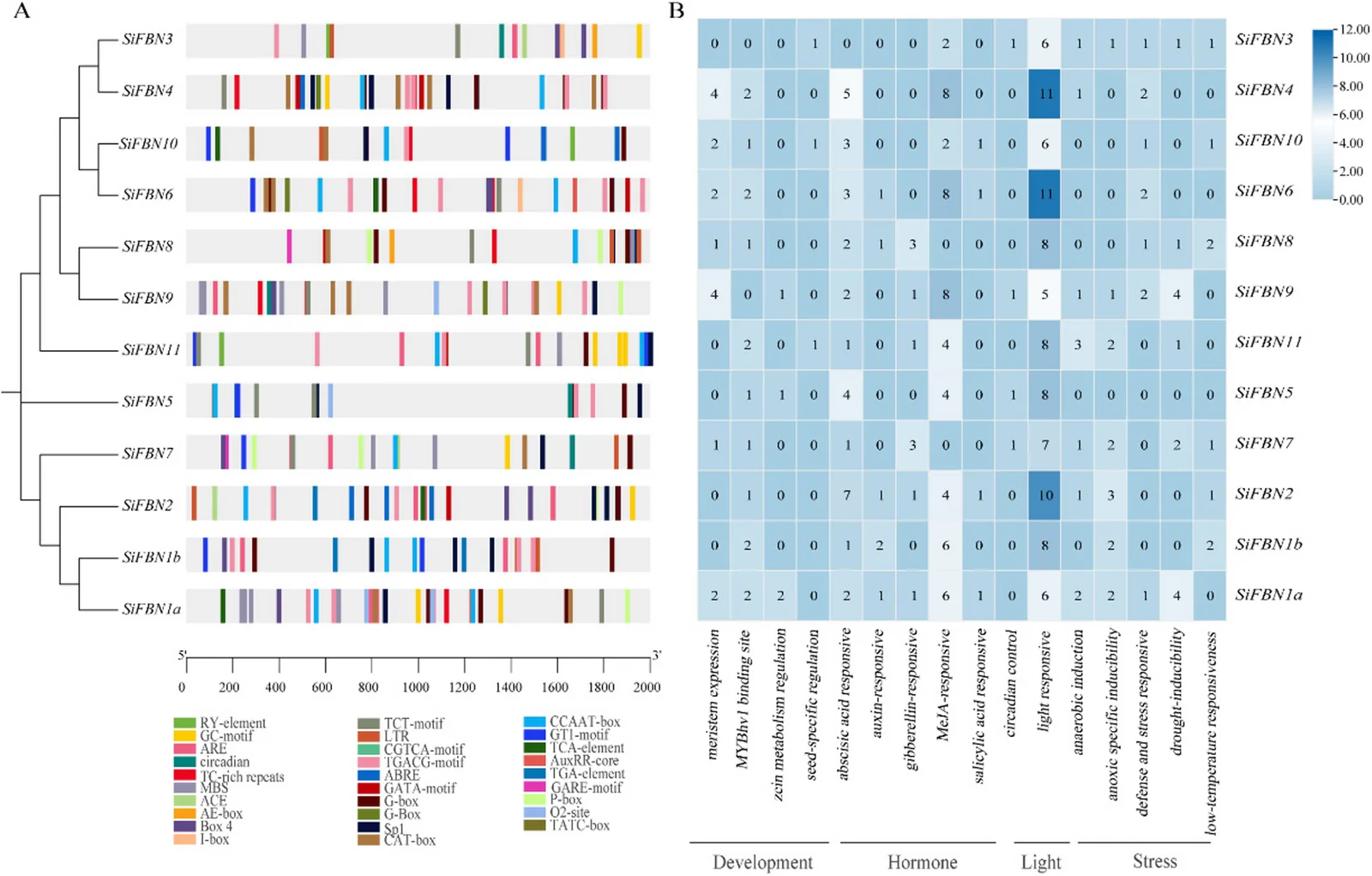
Cis-acting elements in the promoter regions of SiFBN genes. **A** Distribution of cis-acting elements in the 2-kb upstream promoter sequences of 12 SiFBN genes. Different colored boxes represent distinct cis-acting elements, as indicated in the legend below. The phylogenetic tree on the left reflects the evolutionary relationships among SiFBN members. **B** Summary matrix of cis-acting element numbers across SiFBN genes, categorized by functional classes: development (meristem expression, MYB binding site, zein metabolism regulation, seed-specific regulation), hormone (abscisic acid, gibberellin, MeJA, salicylic acid, auxin responsive), light (circadian control, light responsive), and stress (anoxic inducibility, defense and stress, drought inducibility, low-temperature responsiveness). The color scale indicates the number of elements per gene

The promoter regions (2 kb upstream of the transcription start site) of the 12 *SiFBN* genes were analyzed for cis-regulatory elements using PlantCARE. A diverse array of elements associated with growth and development, light responsiveness, hormone signaling, and stress responses was identified (Fig. 3A). In total, 29 distinct element types were predicted. The quantitative distribution of these elements across all family members is summarized in Fig. 3B. Stress-related elements were the most abundant category, with MYB (15 occurrences) and binding sites—key regulators of drought and abscisic acid (ABA) signaling highly enriched. This suggests that *SiFBN* genes may be involved in drought, low-temperature, and biotic stress responses. Light-responsive elements were also prevalent, including 29 G-box elements, multiple GT1-motif, Box 4, and ACE copies. This enrichment aligns with the chloroplast localization of SiFBN proteins and their roles in photoprotection. Hormone-responsive elements included ABRE (ABA-responsive), CGTCA-motif, and TGACG-motif (jasmonic acid and salicylic acid signaling), as well as stress-responsive elements such as STRE and LTR. Growth-related elements (CAT-box, RY-element, O_2_-site) were also detected, implying potential roles in seed maturation and meristem development. Collectively, the complex cis-regulatory landscape of *SiFBN* promoters indicates multifaceted regulatory potential in integrating light, hormone, and stress signals to modulate plastid function and plant adaptation.

### Chromosomal localization and synteny analysis of *SiFBNs*

**Fig. 4:**
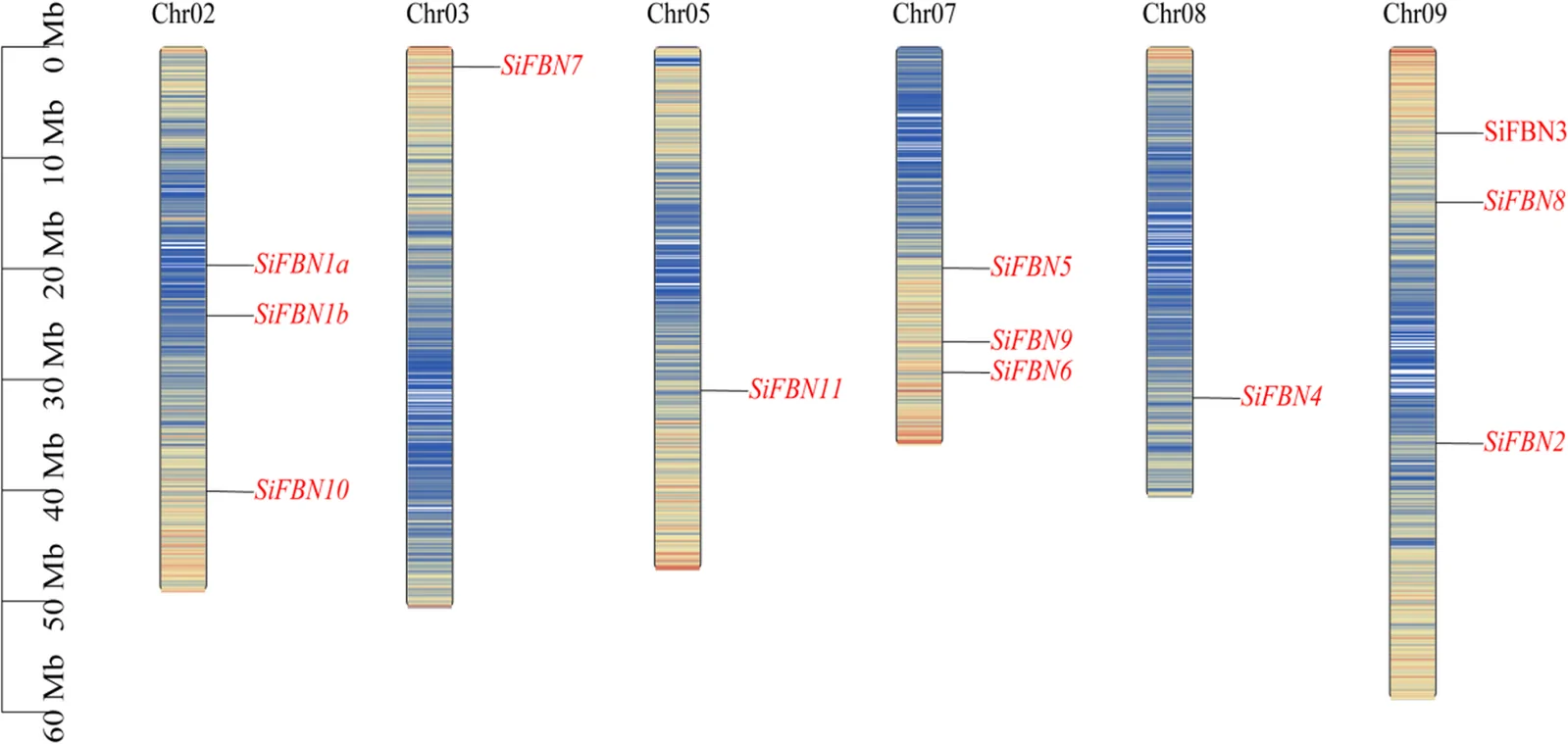
Chromosomal distribution of *SiFBN* genes. Genes are mapped to their respective scaffolds of the foxtail millet genome (v2.2)

**Fig. 5:**
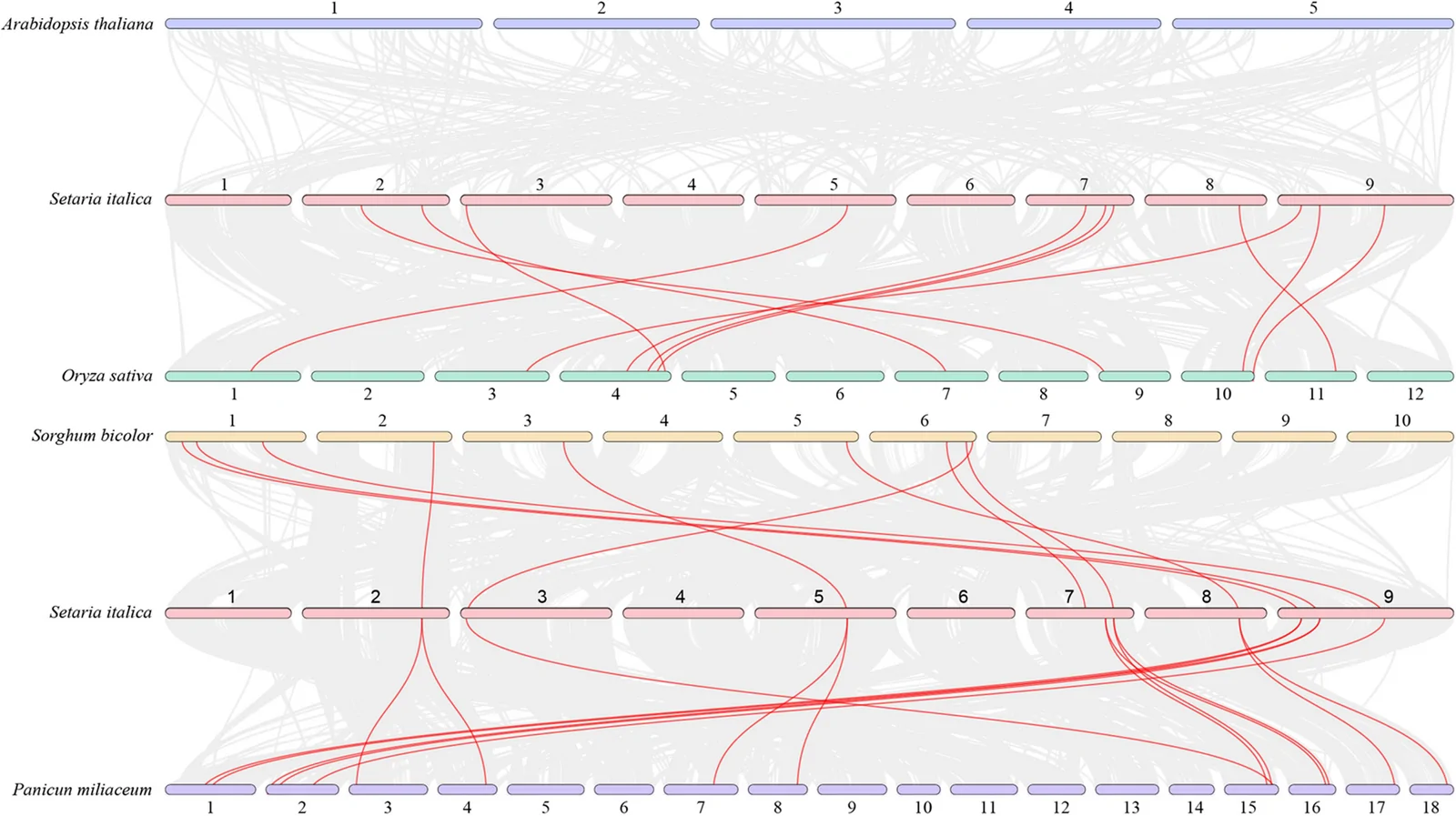
Synteny analysis of *FBN* genes between foxtail millet and other species. Gray lines indicate collinear blocks between genomes, and red lines highlight syntenic FBN gene pairs

Chromosomal localization revealed that the 12 *SiFBN* genes were distributed across six chromosomes (Fig. 4). Chromosomes 2, 7, and 9 each harbored three *SiFBN* genes, while chromosomes 3, 5, and 8 contained only one gene each. To investigate the evolutionary relationships across different species, synteny analysis was performed among *Setaria italica*, *Arabidopsis thaliana*, *Oryza sativa*, *Sorghum bicolo*r, and *Panicum miliaceum* (Fig. 5). No syntenic relationships were detected between *SiFBN* and *AtFBN* genes. Concersely, all *SiFBNs* except for *SiFBN1a* exhibited one or more syntenic genes in rice, sorghum, and broomcorn millet, indicating conserved evolutionary relationships within the Panicoideae subfamily. The absence of synteny for *SiFBN1a* suggests it may represent a lineage-specific duplication in foxtail millet.

### Spatiotemporal and stress-response expression patterns of *SiFBN* family

**Fig. 6:**
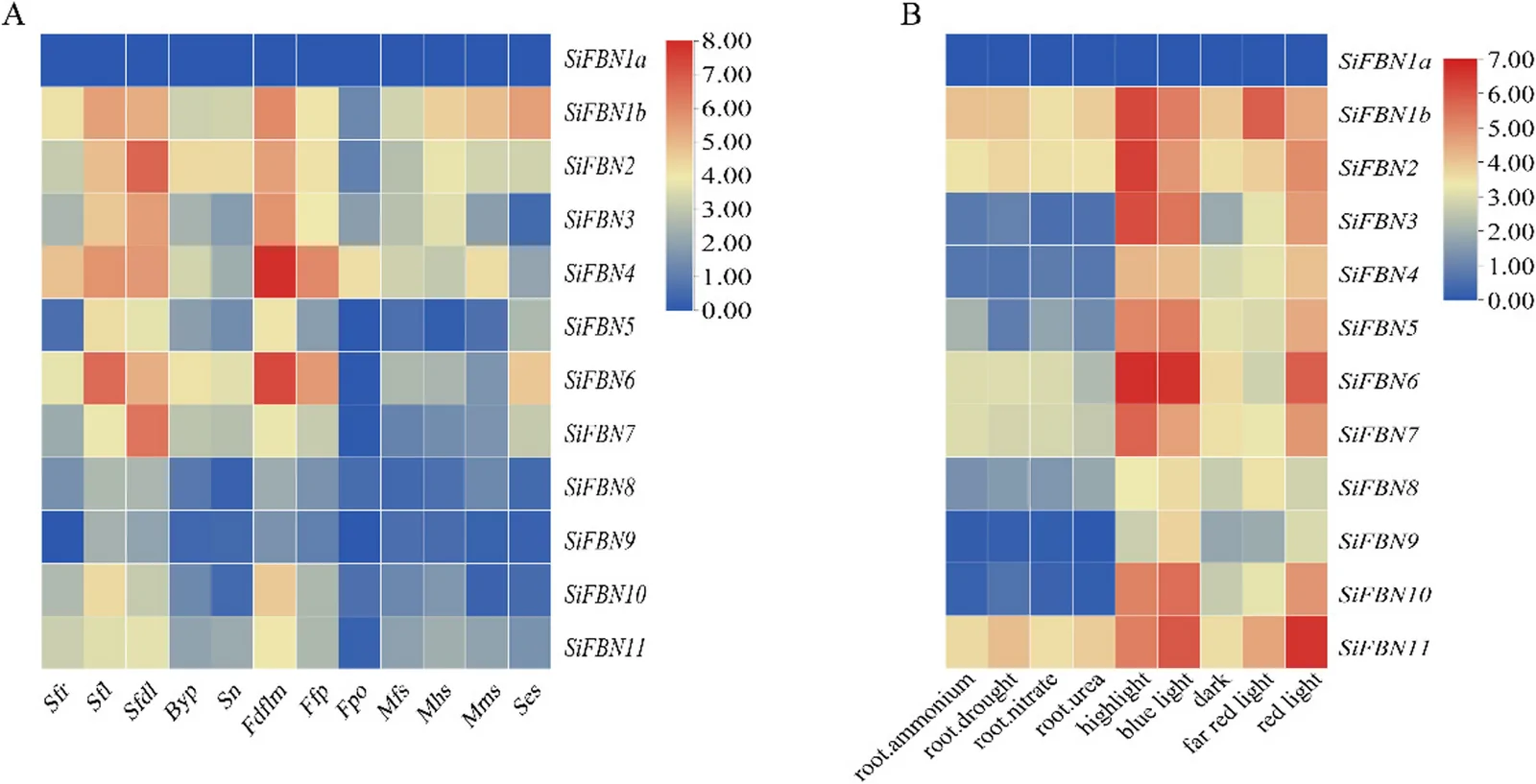
Expression profiles of *SiFBN* genes across different tissues (**A**) and under various abiotic stresses (**B**). Transcript abundance is presented as log_2_-transformed FPKM values

Public available transcriptome data were used to profile *SiFBN* expression across tissues and developmental stages (Fig. 6A) as well as under abiotic stress conditions (Fig. 6B). Pronounced spatiotemporal specificity was observed. *SiFBN1b* and *SiFBN2* were consistently highly expressed in all tissues except pollen, suggesting their housekeeping feature of this two genes. In contrast, *SiFBN1a* expression was negligible (FPKM ≈ 0) across all samples, possibly indicating pseudogenization or condition-specific induction. Most *SiFBN* genes were predominantly expressed in leaves, with levels markedly higher than in roots, consistent with chloroplast localization and roles in photosynthetic tissues. Differential responsiveness to environmental cues was observed. Under root-targeted stresses (nitrogen deficiency, drought), *SiFBN* expression showed limited alteration, suggesting the family is not a primary responder to these root-imposed stresses. Strikingly, nearly all *SiFBN* genes were significantly upregulated under light treatments (red, far-red, blue, and high-intensity light) compared to dark-grown controls. This robust light responsiveness aligns with the abundance of light-regulatory cis-elements in their promoters (Fig. 3) and their predicted chloroplast localization.

### Expression dynamics of *SiFBNs* during grain development and RT-qPCR validation

**Fig. 7:**
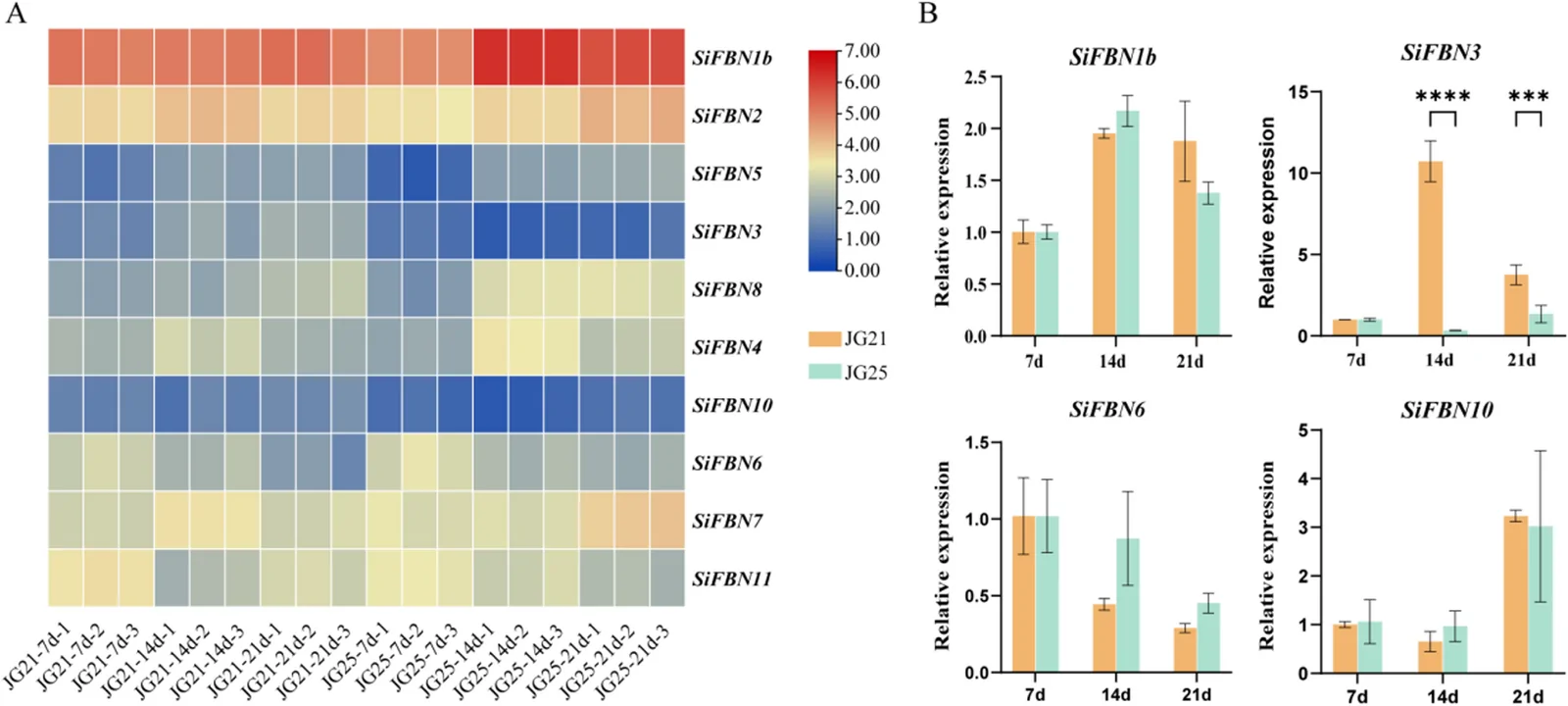
Expression patterns of *SiFBN* genes during grain development in white-grain (JG25) and yellow-grain (JG21) varieties. **A** RNA-seq expression levels (FPKM). **B** RT-qPCR validation. Data are means ± SD of three biological replicates. *P < 0.05, **P < 0.01, ***P < 0.001 (Student’s t-test)

The expression patterns of *SiFBN* genes during grain filling stage were investigated through integrative analysis of RNA-seq data and RT-qPCR validation, which revealed complex spatiotemporal dynamics (Fig. 7). Distinct expression profiles were observed among key family members. Transcriptome analysis indicated that *SiFBN1b* was the most abundantly expressed family member throughout grain development in both JG21 and JG25 (Fig. 7A). RT-qPCR confirmed its high expression level but detected no significant difference between the two varieties at any stage (*P* > 0.05), consistent with a constitutive, non-variety-specific role in this process. *SiFBN3* showed differential expression between the two varieties. Its transcript level was significantly higher in the high-carotenoid variety JG21 than that in low-carotenoid JG25 at 7, 14, and 21 DAA (*P* < 0.01; Fig. 7B). A slight discrepancy in peak timing was noted between platforms (21 DAA in RNA-seq versus 14 DAA in RT-qPCR), which may reflect methodological differences or rapid transcriptional changes during this dynamic phase. Notably, haplotype analysis of 152 accessions revealed no polymorphism in the *SiFBN3* coding or promoter region, suggesting that the observed varietal difference may be attributed to upstream regulatory factors rather than allelic variation at this locus. In contrast, although *SiFBN10* showed differential expression in the RNA-seq dataset, RT-qPCR assay revealed no significant difference between the two varieties (*P* > 0.05). However, pronounced upregulation of *SiFBN10* at 21 DAA was observed in both varieties, pointing to developmentally timed induction during late grain filling. The expression of *SiFBN6* declined progressively across the three developmental stages with no variety-specific difference (*P* > 0.05), indicating regulation primarily by developmental cues. Notably, SiFBN6 clusters phylogenetically with AtFBN6 (Group 6, Fig. 1), a known positive regulator of carotenoid biosynthesis in Arabidopsis, hinting at a possible conserved function. Collectively, these results illustrate the diversity of regulatory strategies employed by the *SiFBN* family during grain development, ranging from constitutive expression (*SiFBN1b*) and differential expression between varieties (*SiFBN3*) to development-controlled expression (*SiFBN6*,* SiFBN10*).

### Subcellular localization of SiFBN3 and SiFBN10

**Fig. 8:**
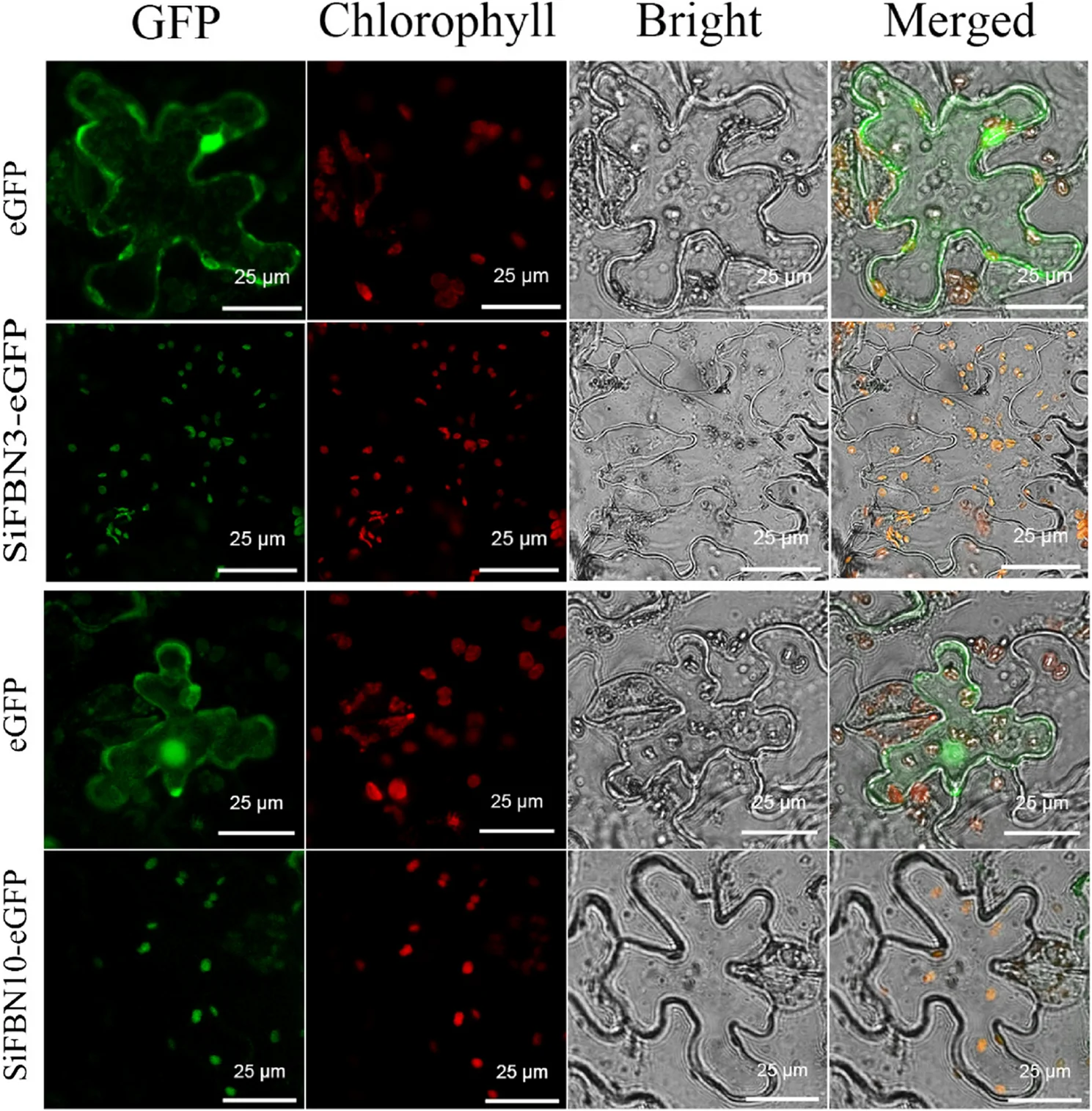
Subcellular localization of SiFBN3 and SiFBN10 in tobacco leaf epidermal cells. GFP fluorescence (green), chlorophyll autofluorescence (red), bright-field image, and merged view are shown for eGFP control, SiFBN₃-eGFP, and SiFBN₁₀-eGFP. Co-localization of GFP with chlorophyll indicates chloroplast localization. Scale bars = 20 μm

To experimentally validate the predicted plastid localization of candidate SiFBN proteins, we performed transient expression assays with SiFBN3-eGFP and SiFBN10-eGFP fusion proteins in *Nicotiana benthamiana* leaf cells. These genes were selected based on their differential expression in transcriptome analysis (cultivar-specific and development-dependent patterns, respectively). Confocal microscopy revealed that the green fluorescent signals of both SiFBN3-eGFP and SiFBN10-eGFP fusion proteins precisely overlapped with the red autofluorescence of chlorophyll (Fig. 8), demonstrating their specific localization to chloroplasts. This confirmation aligns with the in silico predictions and substantiates the potential involvement of *SiFBN3* and *SiFBN10* in chloroplast-localized processes, particularly those associated with plastoglobule function and carotenoid metabolism.

### Differential carotenoid accumulation in yellow and white grained foxtail millet

**Fig. 9:**
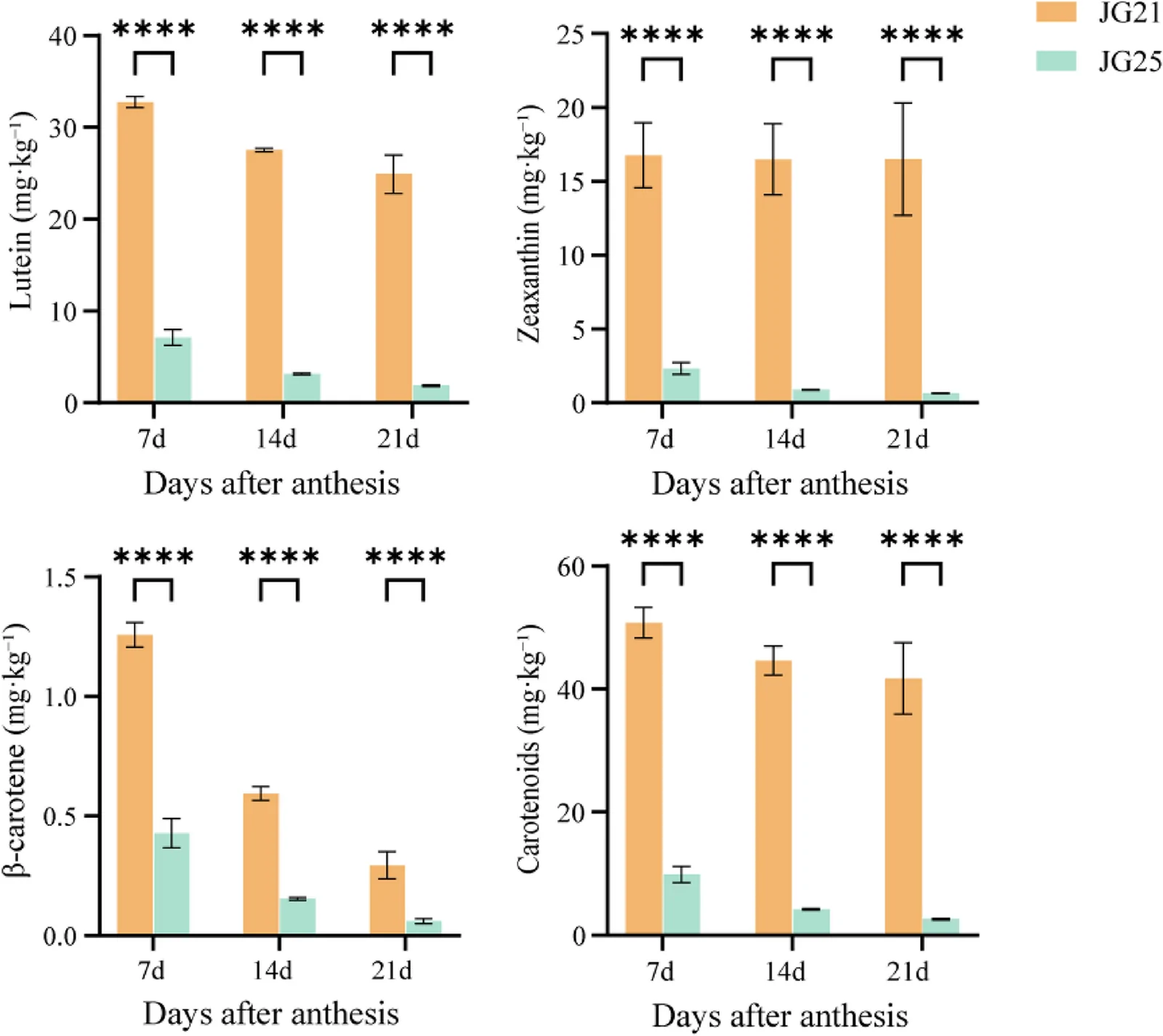
Carotenoid contents in JG21 (yellow-grain) and JG25 (white-grain) at 7, 14, and 21 days after anthesis (DAA). Data are means ± SD of three biological replicates. *P < 0.05, **P < 0.01, ***P < 0.001, ****P < 0.0001 (Student’s t-test between varieties at each time point)

The major carotenoid components (lutein, zeaxanthin, and β-carotene) were quantified in developing grains of the high-carotenoid (JG21) and the low-carotenoid (JG25) varieties using HPLC (Fig. 9). Total carotenoid content, as well as the levels of each individual component, was consistently and significantly higher in JG21 than in JG25 at all three developmental stages examined (7, 14, and 21 DAA). In JG21, carotenoid accumulation peaked at 7 DAA. For instance, lutein reached 32.76 mg·kg⁻¹ at this stage, approximately 4.6-fold higher than that in JG25 (7.12 mg·kg⁻¹). While zeaxanthin levels in JG21 remained relatively stable during grain filling, β-carotene content declined sharply (Supplementary Table S2). By 21 DAA, total carotenoids in JG21 had decreased by 17.83% relative to 7 DAA. This decline was most pronounced for β-carotene (76.65% reduction), whereas lutein and zeaxanthin decreased by 23.95% and 1.45%, respectively. In contrast, all carotenoid components in JG25 declined markedly as development proceeded, with reductions exceeding 70% for each component between 7 and 21 DAA. β-Carotene exhibited the greatest loss (85.56%), resulting in minimal residual levels at maturity.

### Haplotype analysis of differentially expressed genes *SiFBN3* and *SiFBN10*

**Fig. 10:**
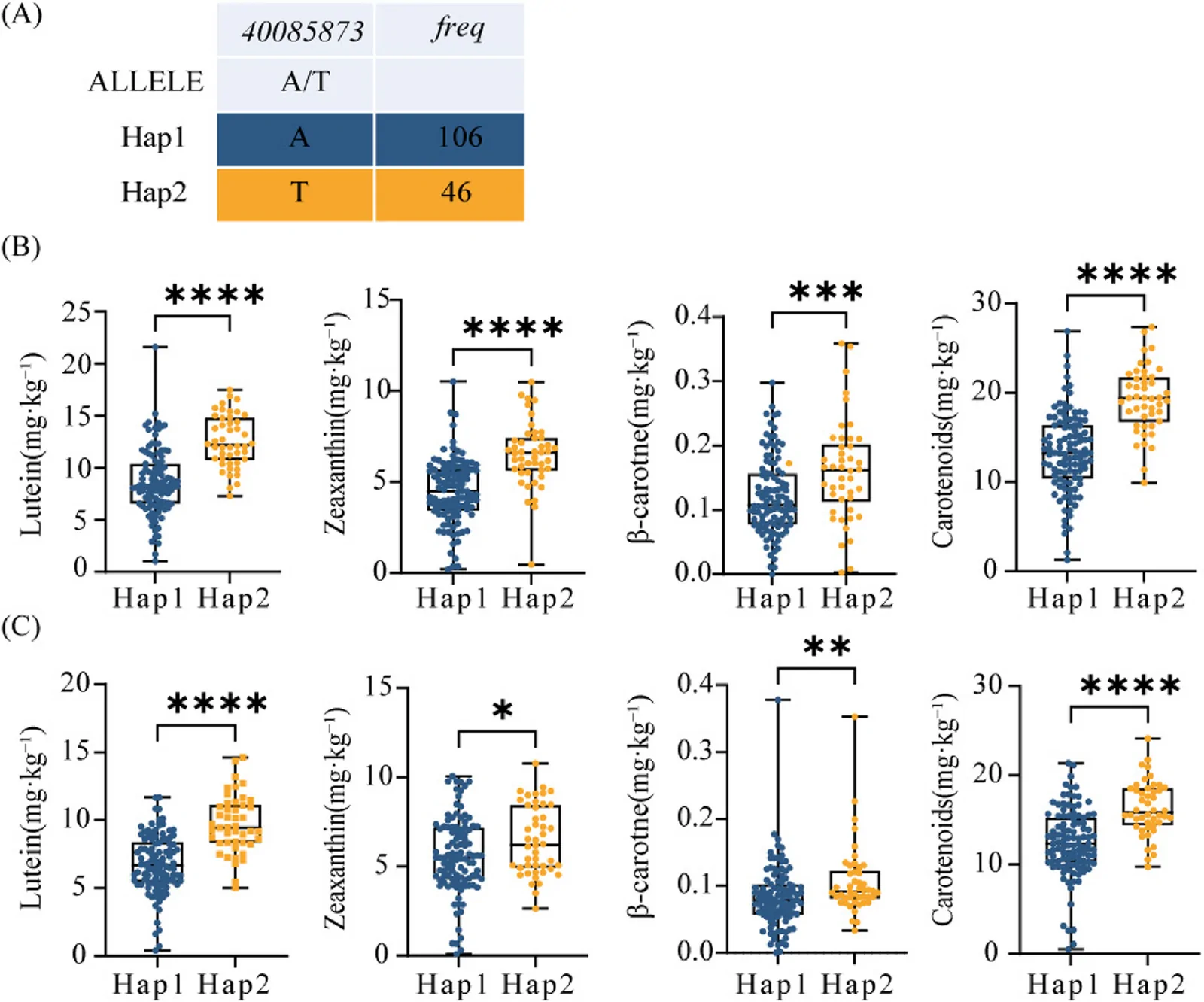
Haplotype analysis of SiFBN10 and its association with carotenoid accumulation. **A** Haplotype distribution of SiFBN10 in the natural population (*n* = 152). The single nucleotide polymorphism (SNP) at position 40,085,873 (A/T) divides the population into two haplotypes: Hap1 (A allele, *n* = 106, 69.7%) and Hap2 (T allele, *n* = 46, 30.3%). **B**, **C** Carotenoid contents of different SiFBN10 haplotypes in 2020 and 2022. Box plots show the distribution of lutein, zeaxanthin, β-carotene, and total carotenoid contents in Hap1 (blue) and Hap2 (orange) accessions. Significant differences were determined by Student’s t-test (***P < 0.001, ****P < 0.0001)

To explore the genetic basis of carotenoid variation, haplotype analysis of candidate genes *SiFBN3* and *SiFBN10* was performed in a natural population of 152 foxtail millet accessions (Fig. 10). The results showed that SiFBN3 exhibited no polymorphism in the natural population, whereas SiFBN10 exhibited two major haplotypes defined by an A/T polymorphism at position 40,085,873 (Hap1: A allele; Hap2: T allele). Hap1 (A allele) was the common haplotype (69.7% frequency), whereas Hap2 (T allele) was the rare haplotype with a frequency of 30.3% (Fig. 10A). Association analysis revealed a strong link between *SiFBN10* haplotypes and carotenoid accumulation. Across two independent growing seasons (2020 and 2022), accessions carrying the Hap2 (T allele) accumulated significantly higher levels of lutein, zeaxanthin, β-carotene, and total carotenoids than those carrying the Hap1 (A allele) (*P* < 0.001; Fig. 10B, C). These results suggest that the A/T variant at the *SiFBN10* locus is associated with carotenoid content variation, where the rare T allele (Hap2) represents the favorable allele. The functional mechanism underlying this association requires further investigation.

## Discussion

### Genomic identification, evolution, and functional divergence of the SiFBN family in foxtail millet

Fibrillin (FBN) proteins are plastid lipid-associated proteins involved in secondary metabolism and abiotic stress responses [33–35]. In this study, we identified 12 *SiFBN* genes in foxtail millet, comparable to rice (11) and sorghum (11), but fewer than Arabidopsis (14). Phylogenetic analysis classified them into 11 subfamilies, consistent with Arabidopsis [36]. Notably, paralogs *SiFBN1a* and *SiFBN1b* clustered in Group I, while the remaining 10 genes distributed across 9 subfamilies, suggesting a recent duplication event followed by subfunctionalization. Gene structure analysis supports functional divergence: exon numbers ranged from 3 (*SiFBN1a*) to 13 (*SiFBN11*), and uneven UTR distribution implies roles for alternative splicing. Notably, *SiFBN11* uniquely contains a Pkc_like superfamily domain in addition to the PAP_fibrillin domain shared by all members, suggesting expanded functional potential. These features indicate that sequence fine tuning may have evolved diverse functions for regulating metabolic pathways or responding to environmental signals [37, 38]. Subcellular localization prediction showed most members targeted to chloroplasts, consistent with FBN functions in plastid lipid metabolism and photoprotection [39].

### Tissue-specific and developmental expression patterns

Public database analysis revealed complex tissue-specific expression of *SiFBN* genes. Most showed high expression in leaves, consistent with chloroplast localization; low expression in roots supports functional concentration in photosynthetic organs, as reported in wheat [19], Arabidopsis [17], and potato [18]. *SiFBN1b* maintained high constitutive expression in all tissues except pollen, potentially serving as a housekeeping gene. In contrast, *SiFBN1a* showed low expression, and whether it represents a pseudogene or is stress-induced remains to be investigated. Under red, far-red, blue, and high-light treatments, most family genes were significantly upregulated, associated with abundant light-responsive cis-elements in their promoters and consistent with FBN roles in light energy utilization [40].

### Candidate gene screening during grain development

To investigate *SiFBN* roles in grain quality, we analyzed expression patterns during grain development in white-grained JG25 and yellow-grained JG21. RNA-seq and RT-qPCR consistently showed *SiFBN3* differentially expressed between varieties, with JG21 significantly higher than JG25 at 7, 14, and 21 DAA, indicating *SiFBN3* participates in carotenoid accumulation through transcriptional regulation. In contrast, *SiFBN10* showed inconsistent patterns between platforms: RNA-seq suggested differential expression, but RT-qPCR showed no significant varietal differences, instead revealing peak expression at 21 DAA. This developmental specificity suggests *SiFBN10* may function during late grain filling in plastoglobule stabilization or pigment sequestration, rather than directly determining varietal differences. Thus, *SiFBN3* is transcriptionally regulated with varietal differences, while *SiFBN10* is developmentally timed without varietal differences, suggesting these two genes regulate carotenoid metabolism through different mechanisms.

### Functional validation: subcellular localization and phenotypic association

To verify functional relevance, we examined subcellular localization using eGFP fusion proteins. Both SiFBN3-eGFP and SiFBN10-eGFP co-localized with chlorophyll autofluorescence in Nicotiana benthamiana epidermal cells, confirming chloroplast localization. This provides critical evidence linking these genes to carotenoid metabolism, as plastoglobules serve as primary carotenoid storage sites. Quantitative analysis revealed significant carotenoid differences between JG21 and JG25, with JG21 higher in lutein, zeaxanthin, β-carotene, and total carotenoids across all stages. The most pronounced relative differences occurred at 21 DAA, coinciding with *SiFBN10* peak expression, suggesting its potential role in late-stage carotenoid accumulation. Longitudinal analysis showed highest absolute content at 7 DAA with varying trends thereafter, indicating complex regulation involving synthesis, storage, and degradation balance. *SiFBN3* varietal expression paralleled pigment differences, while *SiFBN10* peak expression coincided with maximum relative varietal difference, hinting at late-stage pigment stabilization.

### Genetic validation and breeding potential of SiFBN10 haplotypes

To explore the genetic basis of carotenoid variation at the population level, we conducted haplotype analysis of *SiFBN3* and *SiFBN10* using 152 foxtail millet accessions. *SiFBN3* exhibited no sequence polymorphism, indicating strong purifying selection and limited breeding utility through allelic selection. In contrast, *SiFBN10* displayed two distinct haplotypes defined by SNP 40,085,873: Hap2 (T allele, 30.3%, *n* = 46) associated with high carotenoid accumulation; Hap1 (A allele, 69.7%, *n* = 106) associated with low content. Association analysis across two independent years confirmed that Hap2 accessions showed significantly higher lutein, zeaxanthin, β-carotene, and total carotenoids than Hap1 (*P* < 0.001), demonstrating the robustness of this association.

DNA sequencing and cDNA amplification confirmed that the polymorphism at position 40,085,873 is located within the coding sequence of SiFBN10 transcript variant 2. The T allele (Hap2) maintains the canonical TAG stop codon, while the A allele (Hap1) replaces it with AAG (Lys), disrupting normal translational termination. This stop codon loss may result in translational readthrough and C-terminal extension of the protein, although the functional consequences require further validation. Notably, Hap1 predominates in the natural population (69.7%), suggesting that Hap2 represents a rare but agronomically valuable allele for carotenoid biofortification.

The functional divergence between *SiFBN3* and *SiFBN10* reveals distinct strategies for regulating carotenoid metabolism: *SiFBN3* operates through transcriptional dosage regulation with limited allelic diversity, whereas *SiFBN10* provides phenotypic plasticity through a natural polymorphism. This makes SiFBN10-Hap2 particularly attractive for marker-assisted selection, as the T allele can be directly targeted for introgression into elite varieties to enhance grain nutritional quality. Future studies should validate the stop codon polymorphism effects at the protein level and evaluate Hap2 performance across diverse genetic backgrounds and environments to facilitate its deployment in breeding programs.

The temporal discrepancy between carotenoid content (peak at 7 DAA) and SiFBN10 expression (peak at 21 DAA) suggests that SiFBN10 may function primarily in carotenoid sequestration and stabilization during late grain development rather than driving initial biosynthesis. Early biosynthetic genes such as PSY, PDS, and ZDS are likely responsible for the rapid carotenoid accumulation observed at 7 DAA. We acknowledge that our study focused specifically on the FBN family, and the regulatory network governing carotenoid accumulation in foxtail millet likely involves multiple gene families, which warrants further investigation.

## Conclusions

In conclusion, this study systematically identified the FBN gene family in foxtail millet and revealed divergent transcriptional and post-transcriptional regulation of carotenoid accumulation. We suggest that SiFBN3 mediates transcriptional control through differential expression between varieties, while a natural polymorphism in SiFBN10 (A/T at position 40085873) is associated with natural variation in grain carotenoid content. The T allele (Hap2) maintains the canonical stop codon, while the A allele (Hap1) disrupts translational termination. Our findings provide novel insights into the regulatory mechanisms of this nutritionally vital metabolic pathway and deliver a candidate molecular marker for marker-assisted breeding of biofortified foxtail millet varieties.

## Supplementary Information

Supplementary Material 1.

## Data Availability

The raw sequence data reported in this paper have been deposited in the Genome Sequence Archive (GSA: CRA042458) that are publicly accessible at https://ngdc.cncb.org/gsa . The whole-genome resequencing data used for haplotype analysis are available from the corresponding author upon reasonable request due to germplasm resource conservation policies.
