# Supplementary material for: Functional deciphering of the fibrillin gene family in foxtail millet reveals a non-synonymous SNP associated with carotenoid accumulation
Source: BMC Plant Biol. 2026 Aug 11;26:1905. doi: 10.1186/s12870-026-09543-1 (PMC13741731; doi:10.1186/s12870-026-09543-1)
Supplement: Supplementary file 1 [file 12870_2026_9543_MOESM1_ESM.docx]

**Supplementary Table 1 Primer sequences used for RT-qPCR and subcellular localization.**

| Gene name | Forward primer sequence (5'-3') | Reverse primer sequence (5'-3') | Purpose |
| --- | --- | --- | --- |
| SiFBN1a | CAGCCTTGGTTGCAAAGACC | GGTGCTTCCCTCCTTGAACA |  |
| SiFBN1b | ATCGGAACCCCACAACTGAC | TATTTGCAACCGAGGACGCT | Quantitative real-time |
| SiFBN2 | AGGCTCCATTTCTGGACAGC | GGTAGGTTGTCAGGAGCCAC |  |
| SiFBN3 | ATTGGAGGCAGTGAACCAGG | GGCCTCTGTGGTTGCAGTAT |  |
| SiFBN4 | GACGTCGGGCATCAAGATCA | GGTACGTCACCTCGAACTCG |  |
| SiFBN5 | CGTGGACAGGGGGATATTCG | TTCCCCAGGATCGAGATGGT |  |
| SiFBN6 | CTTCTCCTCCGCCATTCCTC | CACTTCCTGGTGCCCATCTT |  |
| SiFBN7 | ATCGTTGGACTTGAACGGCT | CTCTCAAACAAAAGCCGGGC |  |
| SiFBN8 | CTGCGCTTGGTTCAGGAAAC | CGTGTAGCATAGCCTCCAGG |  |
| SiFBN9 | GCAGCTGGGAGGTACCTTTT | TCCTTGGCAACCCGTATGTC |  |
| SiFBN10 | GCCGGTCTCAAGGACTTTCA | ACTGCACACCACCAACTTGA |  |
| SiFBN11 | AGCAAGTAACGGCGAAAGGA | ACGCAAACAGTATGGGTCGT |  |
| SiActin | AACATTGTGCTCAGCGGTGG | TGGAAGGTGCTAAGGGAGGC |  |
| SiFBN3-eGFP | TGGAGAGAACACGGGGGACTCTT  GACCATGGCGCCCGCCTCGCTCA  TGCTCTCTCTCCCGCGCGCG | CTGAAGATGGTCGACCCAGCAT  ACAGAGTTCCATTGACTAGTAA  AGGAGAAGAACTTTTCACTGGAG | Subcellular localization |
| SiFBN10-eGFP | TGGAGAGAACACGGGGGACTCT  TGACCATGGCCGCCTCCGCAGCA  GCAG CCGGAGGCATCGTCCA | CTCCAGTGAAAAGTTCTTCTCCTT  TACTAGTCGTTGCATTTGCGAT  GCGTAA GTGGACTA |  |

**Supplementary Table 2 Significance of differences in carotenoid content among developmental stages within each variety (JG21 and JG25).**

|  | Lutein | | Zeaxanthin | | β-carotene | | Carotenoids | |
| --- | --- | --- | --- | --- | --- | --- | --- | --- |
|  | JG21 | JG25 | JG21 | JG25 | JG21 | JG25 | JG21 | JG25 |
| 7DAA | 32.75±0.59a | 7.12±0.87a | 16.77±2.20a | 2.33±0.39a | 1.23±0.05a | 0.43±0.06a | 50.77±2.49a | 9.88±1.30a |
| 14DAA | 27.53±0.19b | 3.16±0.10b | 16.50±2.41a | 0.89±0.03b | 0.59±0.03b | 0.15±0.01b | 44.63±2.35b | 4.21±0.12b |
| 21DAA | 24.91±2.09b | 1.88±0.19c | 16.52±3.81a | 0.66±0.03c | 0.29±0.06c | 0.06±0.01c | 41.73±5.82b | 2.60±0.11c |

**Data are presented as mean ± SD (n=3). Different lowercase letters within a column indicate significant differences among developmental stages (7, 14, 21 DAA) for the same material and carotenoid component, as determined by one-way ANOVA followed by Tukey's honestly significant difference (HSD) test (P < 0.05).**
